# Impact of Corn Starch Molecular Structures on Texture, Water Dynamics, Microstructure, and Protein Structure in Silver Carp (*Hypophthalmichthys molitrix*) Surimi Gel

**DOI:** 10.3390/foods13050675

**Published:** 2024-02-23

**Authors:** Congyun Jiang, Xin Yang, Songyi Lin, Yumeng Yang, Jinzhi Yu, Xinqi Du, Yue Tang

**Affiliations:** 1 SKL of Marine Food Processing & Safety Control, School of Food Science and Technology, Dalian Polytechnic University, Dalian 116034, China; cy__jiang@163.com (C.J.); yxabc126@163.com (X.Y.); linsongyi730@163.com (S.L.); yym15641600501@163.com (Y.Y.); jinzhiyu0118@163.com (J.Y.); dxq0114@163.com (X.D.); 2 National Engineering Research Center of Seafood, Dalian Polytechnic University, Dalian 116034, China; 3 Collaborative Innovation Center of Seafood Deep Processing, Dalian Polytechnic University, Dalian 116034, China; 4 Engineering Research Center of Special Dietary Food, The Education Department of Liaoning Province, Dalian 116034, China; 5 Engineering Research Center of Food, The Education Department of Liaoning Province, Dalian 116034, China

**Keywords:** corn starch molecular structure, molecular weight correlation, surimi gel properties, protein network formation

## Abstract

This study systematically investigates the impact of corn starch molecular structures on the quality attributes of surimi gel products. Employing molecular analyses to characterize corn starch, three amylopectin fractions (A, B_1_, and B_2_), categorized by the degree of polymerization ranges (6 < X ≤ 12, 12 < X ≤ 24, and 24 < X ≤ 36, respectively) were specifically focused on. The surimi gel quality was comprehensively assessed through texture profile analysis, nuclear magnetic resonance, scanning electron microscopy, stained section analysis, and Fourier transform infrared spectroscopy. Results indicated the substantial volume expansion of corn amylopectin upon water absorption, effectively occupying the surimi gel matrix and fostering the development of a more densely packed protein network. Starch gels with higher proportions of A, B_1_, and B_2_ exhibited improved hardness, chewiness, and bound water content in the resultant surimi gels. The weight-average molecular weight and peak molecular weight of corn starch showed a strong positive correlation with surimi gel hardness and chewiness. Notably, the secondary structure of proteins within the surimi gel was found to be independent of corn starch’s molecular structure. This study provides valuable insights for optimizing formulations in surimi gel products, emphasizing the significance of elevated A, B_1_, and B_2_ content in corn starch as an optimal choice for crafting dense, chewy, water-retaining surimi gels.

## 1. Introduction

Surimi-based products are renowned for their elasticity and nutritional benefits, featuring high protein and low-fat content. This diverse category includes popular items such as kamaboko, fish balls, fish sausage, chikuwa, and crab sticks. The global appeal of these products is attributed to their nutritional richness, enticing flavor, and resilient texture. Silver carp (*Hypophthalmichthys molitrix*) is increasingly being explored as a cost-effective alternative to sea fish in surimi production due to its swift growth and high yield [1]. However, being a freshwater fish, silver carp exhibits weaker gelling capabilities compared to marine counterparts, thereby limiting its processing and utilization [2]. To elevate the quality of freshwater fish surimi gels, additional ingredients like starch, salt, and water are commonly introduced [3]. Starch additives offer three main functional advantages in surimi-based products: improved gel performance and sensory quality, increased yield due to high-water absorption and swelling ability, and reduced production costs [4]. Numerous studies have concentrated on optimizing starch types to enhance surimi gel quality. For instance, surimi-starch gels incorporating native potato starch demonstrated superior hardness, chewiness, whiteness, and gel strength compared to those with corn starch [5]. Variations in properties were also noted in surimi gels containing different starch sources, such as potato and wheat starches [6], and correlated with amylose and amylopectin content [7]. While existing studies emphasize the relationship between starch type, granule size, amylose to amylopectin ratio, and surimi gels properties, a comprehensive understanding of the molecular structure of starch and its impact on surimi gels is lacking.

Starch, a polymeric carbohydrate composed of glucose units linked by glycosidic bonds, encompasses two main types: amylose and amylopectin. Amylose is characterized by a relatively elongated and less branched structure, whereas amylopectin exhibits high branching [8]. The molecular structure of starch, specifically the chain-length distributions (CLDs), is a critical parameter for evaluating corn starch properties. Amylopectin is classified into A (6 < X ≤ 12), B_1_ (13 < X ≤ 24), B_2_ (24 < X ≤ 36), and B_3_ (37 < X ≤ 100). Similarly, amylose CLDs are categorized into short (100 < X ≤ 1000), medium (1000 < X ≤ 5000), and long amylose chain regions (5000 < X ≤ 20,000) [9]. Recent research emphasizes the pivotal role of starch molecular structure in influencing a range of properties, including physicochemical aspects, food properties, and the quality of starch processing products [10,11,12]. Importantly, starch, akin to other thickeners/gums utilized in the food industry, significantly contributes to enhancing the texture, physical attributes, and chemical properties of food products. For instance, the incorporation of thickeners, including starch, has proven to notably improve the deformation resistance capability of ink formulations [13]. Similarly, the addition of gums to protein systems has demonstrated enhancements in rheological properties and water-holding capacity [14].

This study aims to explore how corn starch molecular structures influence various aspects of surimi gels, including texture properties, water mobility, microstructure, and associated mechanisms. By analyzing whole corn starch molecular size distributions and chain-length distributions (CLDs), the relationship between starch molecular structure and properties exhibited by surimi gel was established by correlation analyses. The findings contribute valuable insights into enhancing surimi gel products by strategically adjusting starch molecular structure, providing a green and sustainable approach to improving product properties.

## 2. Materials and Methods

### 2.1. Materials

Low amylose content corn starch (LACCS) with 6.44% amylose content was sourced from Cofco Co., Ltd., Beijing, China. High amylose content corn starch (HACCS) with 69.89% amylose content was purchased from Quanyin Xiangyu Biotechnology Co., Ltd., Beijing, China. Silver carp surimi (SCS), characterized by a moisture content of 70.05%, was sourced from Anjing Food Group Co., Ltd., Xiamen, China. Other chemical reagents employed adhered to analytical grade standards.

### 2.2. Sample Preparation

The process involved the mixing of LACCS and HACCS in varying mass ratios: 0:10, 2:8, 4:6, 6:4, 8:2, and 10:0, denoted as NG010, NG28, NG46, NG64, NG82, and NG100, respectively. The resulting mixed starch served as the basis for subsequent starch index assessments.

The process commenced by gradually thawing raw surimi blocks at 4 °C overnight until achieving a semi-thawed state. Following cutting into approximately 1 cm cubes, a food processor (QSJ-D02Q1, Bear Electric Co., Ltd., Shunde, China) blended the surimi at 4500 rpm for 2 min. The addition of NaCl (2%) occurred, and blending persisted for an additional 2 min. Subsequently, various mixed starch compositions with a 12% addition (0:10, 2:8, 4:6, 6:4, 8:2, and 10:0, respectively) and ice water were blended with surimi at 4500 rpm for 2 min, adjusting the surimi moisture content to 78%. Throughout the entire process, the temperature was maintained at less than 10 °C. The blended surimi was then packed into polypropylene plastic casings (Φ = 25 mm, h = 30 mm) and tightly sealed at both ends. Finally, surimi gels (NG010, NG28, NG46, NG64, NG82, and NG100) were obtained using a two-step heating method: 60 min at 40 °C followed by 30 min at 90 °C. The preparation of the blank control check group (CK) followed the same steps, excluding the addition of starch. All samples underwent immediate cooling in ice water for 30 min and were stored at 4 °C overnight for subsequent analysis.

### 2.3. Molecular Weight Distribution

Following established research methodologies [15], a 5 mg starch sample underwent a meticulous blending process with a 5 mL solution of DMSO containing LiBr (0.596 *w*/*w*) (DMSO/LiBr). This amalgamation was subjected to heating at 80 °C for 3 h using a thermomixer. To evaluate weight-average molecular weight (Mw) and peak weight molecular weight (M_p_), various fractions underwent scrutiny through size-exclusion chromatography with a DAWNR HELEOSTM-II laser photometer (He-Ne laser, λ = 663.7 nm, Wyatt Technology Co., Santa Barbara, CA, USA), accompanied by refractive index detection (Optilab T-rEX, Wyatt Technology Co., Santa Barbara, CA, USA) equipped with three tandem columns (300 × 8 mm, Shodex OHpak SB-805, 804, and 803; Showa Denko KK, Tokyo, Japan) maintained at 60 °C. The dn/dc value of the fractions in the DMSO solution was determined to be 0.07 mL/g. The entire data acquisition and processing were undertaken using ASIRA 6.1 (Wyatt Technology Inc., Waltham, MA, USA).

### 2.4. Chain-Length Distributions (CLDs) of Debranched Starch

To ascertain the relative molecular weight distributions of debranched starch, a high-temperature gel-permeation chromatography (GPC) system (PL-GPC 220, Polymer Laboratories Varian, Inc., Amherst, MA, USA) was employed, following the methodology outlined by Pan et al. [16]. The GPC results were analyzed with a focus on amylopectin (AP), amylopectin short chains (AP_1_), amylopectin long chains (AP_2_), and amylose chains (AM). Calculations for amylopectin chain distributions (DP 6–12, DP 13–24, DP 25–36, and DP > 37) were calculated based on the amylopectin cluster model [17].

### 2.5. Amylose Content, Amylopectin Content, and AM/AP of Starch

The determination of amylose content in starch followed the methodology outlined in the China National Standards (GB/T 15683-2008) [18]. Subsequently, the amylopectin content and the ratio of amylose to amylopectin (AM/AP) for corn starch were calculated using the following equations:

Amylopectin content (%)=1−Amylose content (%)

$$AM/AP=\frac{Amylose\;content\;(\%)}{Amylopectin\;content\;(\%)}$$

### 2.6. Dynamic Rheological Measurement

The rheological characteristics of silver carp surimi samples were analyzed using a rotational rheometer (TA-DHR2, Waters Corporation, New Castle, DE, USA) equipped with a parallel plate geometry measuring system. All the measurements were conducted at 25 °C. To prevent dehydration of surimi samples during testing, a thin layer of silicone oil was applied to the sample edges. The dynamic viscoelastic properties were assessed at a 1% strain (within the linear viscoelastic region) and a frequency range of 0.1–100 rad/s. Both the storage modulus (G′) and loss modulus (G″) were meticulously recorded [19].

### 2.7. Textural Profile Analysis (TPA)

Silver carp surimi gels were analyzed for textural properties using the TPA method, as described by Zhao et al. [20], with minor modifications. The assessment of textural parameters, including hardness, springiness, cohesiveness, and chewiness, was conducted employing a texture analyzer (TA-XT Plus, Stable Micro Systems, London, UK). Operating conditions for the analyzer included a probe model P50, compression ratio of 50%, interval time of 5 s, and trigger force of 5 g. Pretest speed, test speed, and posttest speeds were 2 mm/s, 1 mm/s, and 2 mm/s, respectively. To ensure reliability and consistency, each sample set underwent three parallel tests.

### 2.8. Water-Holding Capacity (WHC)

To determine the WHC of the surimi/starch composite gels, a slightly modified method from previous reports was employed [21]. Briefly, the weight of the surimi/starch composite gels (2.00 g) was initially measured (W_1_). Subsequently, the gels underwent centrifugation at 3600× *g* for 10 min (25 °C), placed in a centrifuge tube, and coated with double layers of filter paper. Following the removal of surface moisture from the gels, the samples were reweighed (W2). The WHC was then calculated by the provided equation:

$$WHC\;(\%)=(1-\frac{W_{1}-W_{2}}{W_{1}\times 78\%})\times 100$$

where 78% is the total moisture content of surimi gels.

### 2.9. LF-NMR Spin-Spin Relaxation (T_2_) Measurement

The assessment of moisture distribution and relaxation time involved utilizing a Niumag Pulsed NMR analyzer (Me-soMR23-060H-I, Niumag Co., Ltd., Suzhou, China). This method, with minor adjustments, followed the protocol outlined by Sun et al. [22]. Cylindrical wafers (Φ = 25 mm, h = 5 mm) were meticulously crafted from silver carp surimi gels, and the Carr–Purcell–Meiboom–Gill (CPMG) pause sequence was employed to measure transverse relaxation (T_2_). Each sample underwent three scans, and all measurements were carried out at 25 °C.

### 2.10. Distribution and Morphology of Gels

The assessment of starch distribution and morphology within the surimi gel matrix employed a well-established approach utilizing periodic acid-Schiff/naphthol yellow S double staining [23]. In a comprehensive procedure, surimi/starch composite gels underwent immersion in a 10% formalin solution to fix the structure. Subsequent steps included dehydration, paraffin embedding, and the preparation of 8-μm-thick serial sections using a precision microtome (RM-2016, Leica Co., Ltd., Witzler, Germany). Following deparaffinization with xylene, these sections were stained with periodic acid-Schiff and naphthol yellow S, representing starch in purple and protein in yellow. A light microscope (Eclipse CI, Nikon, Tokyo, Japan) equipped with a 35 mm photomicrography camera (Microflex HFX-IIA, Nikon, Tokyo, Japan) was then employed for a detailed examination of starch distribution in the composite gels.

### 2.11. Cryo-Scanning Electron Microscopy (Cryo-SEM)

The microstructures of silver carp surimi gels, incorporating starch with distinct molecular structures, were meticulously examined using a cryo-SEM (SU8010, Hitachi, Japan). The sample, carefully embedded in a sample tray, underwent cryo-preparation within the specialized system (PP3010T, Quorum Inc., East Sussex, UK) containing liquid nitrogen sludge, ultimately being flash-frozen to an extreme temperature of −180 °C. Following the controlled sublimation process, the specimens were meticulously gold-plated under an argon atmosphere. Employing cryo-SEM with an accelerating voltage of 10 kV, high-resolution images were acquired to unveil the intricate microstructural details [24].

### 2.12. Fourier Transform Infrared (FT-IR) Spectroscopy

The surimi gels underwent freeze-drying and were subsequently ground into a fine powder. A 1.5 mg portion of the powder was meticulously mixed with 150 mg of KBr, pressed into a transparent sheet, and analyzed using a Nicolet iS5 FT-IR spectrometer (Thermo Fisher Instruments Co., Ltd., Shanghai, China). The samples underwent 32 scans across the 400 to 4000 cm^−1^ range, with a resolution set at 4 cm^−1^ [25]. Relative intensity analysis was performed using both OMNIC 9.2 software (Thermo Fisher Scientific Inc., Waltham, MA, USA) and PeakFit 4.12 software (Systat Software Inc., San Jose, CA, USA).

### 2.13. Statistical Analysis

All experimental procedures were conducted in triplicate, and the experimental data are expressed as mean ± standard deviation. Statistical analysis involved the application of analysis of variance (ANOVA) to evaluate the significance of the collected data. Post-hoc testing using Duncan’s test was then carried out through SPSS 19.0 software (IBM Inc., Armonk, NY, USA) to identify any significant differences between datasets, with a *p*-value < 0.05 considered noteworthy. Pearson correlation analysis was then employed to explore the relationships between starch molecular structure and a range of parameters, encompassing texture characteristics, rheological properties, moisture distribution, and protein conformation within the silver carp surimi gels.

## 3. Results

### 3.1. Molecular Structure of Starch

**Figure 1:**
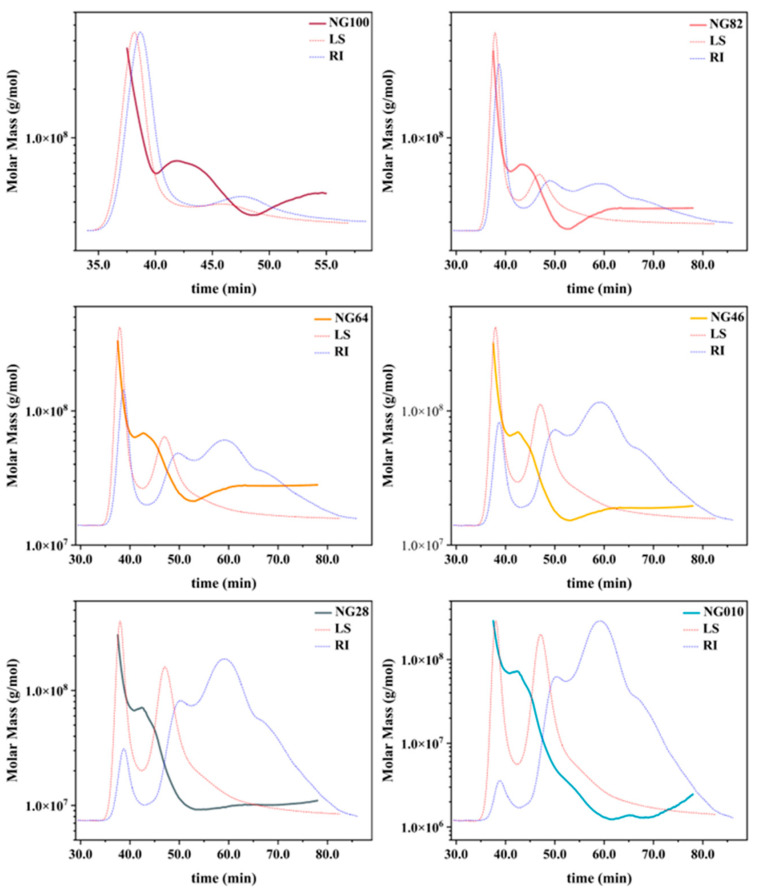
SEC molecular mass distribution and molar mass of whole starch samples. The red dotted line (LS) represents the multi-angle laser light scattering signal, reflecting the molecular size of samples, while the blue dotted line (RI) indicates the differential signal, reflecting sample concentration. NG010, NG28, NG46, NG64, NG82, and NG100 denote the mixed starch samples with mass ratios of LACCS and HACCS at 0:10, 2:8, 4:6, 6:4, 8:2, and 10:0, respectively.

In Figure 1 and Table 1, a discernible diversity emerges in M_w_ and M_p_ among different corn starch samples. M_w_ values range from 1893.79 to 65,304.72 kDa, while M_p_ values span from 1331.81 to 113,771.44 kDa. The significant differences in M_w_ and M_p_ values among the corn starch samples can be attributed to variations in types and mixed mass ratios.

**Figure 2:**
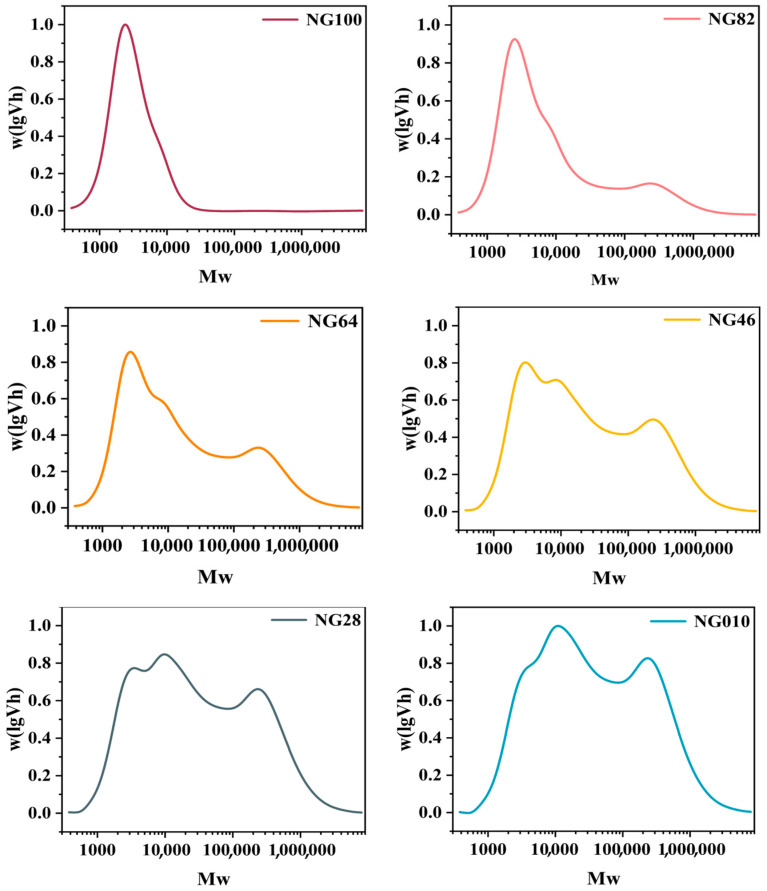
Gel-permeation chromatography results of the chain-length distribution (CLDs) in whole starch samples. NG010, NG28, NG46, NG64, NG82, and NG100 represent the mixed starch samples with mass ratios of LACCS and HACCS at 0:10, 2:8, 4:6, 6:4, 8:2, and 10:0, respectively.

As illustrated in Figure 2 and Table 1, a significant difference in CLDs profiles was evident among all starch samples. Particularly, HACCS exhibited the lowest proportions of A, B_1_, and B_2_ chains but the highest percentages of B_3_, short amylose chain regions, medium amylose chain regions, and long amylose chain regions. In contrast, LACCS displayed the opposite trend. The A and B_1_ chains, forming a double helix and crystal cluster, represent the outermost chains of amylopectin, influencing crystal structure and crystallinity. This suggests that HACCS has the highest amylopectin content [26]. However, the ratio of AM area to the total area, indicative of true amylose content, was notably lower than the apparent amylose contents measured using iodine colorimetry (6.44–69.89%, Table 1). This discrepancy is attributed to the iodine binding capacity of the long branch chains of amylopectin (DP ≥ 60 in B_3_) [27].

### 3.2. Rheological Properties of the Surimi-Starch System

**Figure 3:**
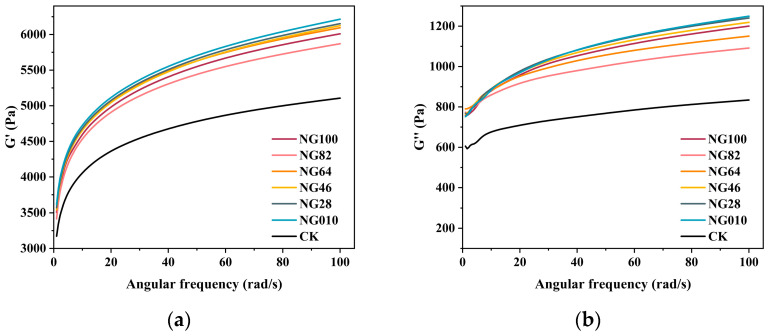
The G’ (**a**) and G’’ (**b**) against angular frequency profiles of surimi/starch composites. NG010, NG28, NG46, NG64, NG82, and NG100 denote surimi/starch composite gels with added mixed starch at mass ratios of LACCS and HACCS at 0:10, 2:8, 4:6, 6:4, 8:2, and 10:0, respectively.

The rheological properties of the surimi-starch system were assessed using the storage modulus (G′) and the loss modulus (G′′). G′, indicative of resistance to elastic deformation in an elastic solid, consistently exceeded G′′ within the linear viscoelastic region for all the surimi-starch system samples (Figure 3a,b). This highlights the capacity of starch gels to form a predominantly or gel-like structure, emphasizing an overall elastic-dominant state in the entire gel system [28]. Upon the addition of starch, both G′ and G′′ values for all surimi-starch system samples surpassed those of surimi alone (CK). Notably, the surimi-starch system incorporating NG010 exhibited the highest G′ and G′′ values, likely attributed to its elevated amylose content. This observation is in agreement with findings from another study [7], affirming a positive correlation between amylose content and the values of G′ and G′′. The rheological analysis results underscore the suitability of all surimi-starch systems for subsequent surimi gel development.

### 3.3. Textural Properties of the Surimi Gel

TPA stands as a widely utilized method in food studies, providing crucial insights into the mechanical properties during substantial deformation [29]. Table 2 encapsulates the TPA parameters of surimi gels, encompassing hardness, springiness, cohesiveness, and chewiness. The addition of starch led to a significant increase in the texture parameters (hardness, springiness, and chewiness) compared to the control sample, particularly in the NG82 group (*p* < 0.05). This phenomenon aligns with previous research highlighting the role of starch in contributing to a denser network structure of surimi gel, consequently increasing the hardness and chewiness [30]. Additionally, starch addition led to a reduction in cohesiveness [31]. The ratio of LACCS to HACCS transitioning from 0:10 (NG010) to 8:2 (NG82) reinforced hardness, springiness and chewiness. However, a further increase in the LACCS to HACCS ratio to 10:0 (NG100) resulted in a decline in these textural parameters. This variation can be due to the fact that, as the ratio increased to 8:2 (NG82), LACCS acted as a filler, strengthening the surimi gel network. However, with a subsequent increase in LACCS and a continued decrease in HACCS, excessive LACCS could not inhibit the diminishing surimi gel formation ability, leading to reduced gel cohesiveness (*p* < 0.05). These dynamics are contingent on the type of additives used and the specific meat species [32]. The distinctive types and molecular sizes of HACCS and LACCS result in varied interactions with protein, exerting a significant impact on the texture properties of silver carp surimi gel (*p* < 0.05).

### 3.4. Water-Holding Capacity of the Surimi Gel

WHC serves as a critical quality indicator, reflecting the gel’s ability to effectively bind water [33]. A higher WHC value indicates increased water retention in surimi gels. The WHC values for surimi gels with varying ratios of LACCS to HACCS are presented in Table 3. In comparison to the WHC of CK (69.50%), the addition of starches elevated the WHC (78.86–93.16%) of surimi gel. This enhancement was attributed to the starch’s water-absorbing properties and the formation of a porous gel network induced by starch incorporation, facilitating increased water entrapment in the surimi gel matrix [4]. Notably, WHC exhibited variations with changes in the ratio of LACCS to HACCS. Specifically, WHC heightened with the proportion of LACCS, influenced by the distinct water-absorbing characteristics of corn amylopectin. Previous studies suggested that the water absorption and expansion rate of amylopectin surpass those of amylose at temperatures ≥ 65 °C. This implies that samples with high amylopectin content demonstrate a heightened water retention capacity, absorbing water within the surimi gel [34].

### 3.5. LF-NMR Analysis of the Surimi Gel

**Figure 4:**
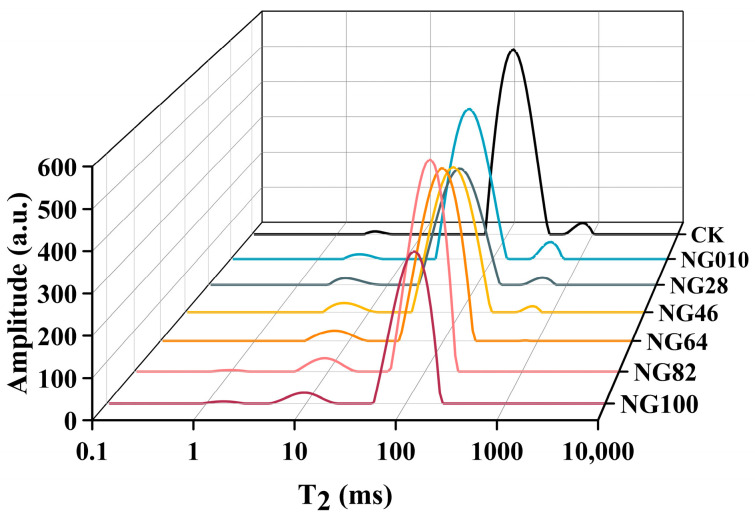
Distribution curves of T_2_ relaxation time for different surimi/starch composite gels. NG010, NG28, NG46, NG64, NG82, and NG100 represent surimi/starch composite gels with added mixed starch at mass ratios of LACCS and HACCS at 0:10, 2:8, 4:6, 6:4, 8:2, and 10:0, respectively.

LF-NMR analysis allows for the non-destructive examination of relaxation time (T_2_), providing insights into the mobility and distribution of various water molecules within the surimi gel network [35]. The T_2_, discerned through the inversion curve for water distribution, unveils peaks at 1–10 ms (T_21_), 10–100 ms (T_22_), and 100–1000 ms (T_23_), representing bound water, immobile water, and free water, respectively [36]. Peak areas, denoted as A_21_, A_22_, and A_23_, offer a reflection of the distribution of distinct water forms. Figure 4 and Table 3 showed the continuous distribution curves of T_2_ in surimi gels, including peak time and areas. Remarkably, T_21_ decreased in all surimi-starch gels versus compared to CK, signifying an augmented binding between the gel and water following starch addition [36]. Furthermore, T_22_ in surimi/starch composite gels (54.88–120.41 ms) was shorter than that in the pure surimi gel (123.22 ms), suggesting that starches enhanced the gel’s ability to immobilize more water, restricting the free movement of water molecules and thereby improving water retention in the composite gel system [36]. This finding aligns with the higher WHC results presented earlier in Table 3. Significantly, the peak area of A_22_ covered a substantial portion (97.22%) of the total peak area, contrasting with less than 2.66% for A_23_ and 0.60% for A_21_. This dominance of immobile water was consistent with prior research [37], indicating that immobile water prevails [38]. Upon adding starches, the proportion of water molecules in A_21_ significantly increased, resulting in a decrease in A_22_ and A_23_ proportions. This suggested that part of the free water and immobile water in gels transformed into bound water, and the water mobility of surimi gels was reduced due to the water-absorption ability of starch [36]. However, this trend became more pronounced with the increase in LACCS in the surimi-starch composite gels. Research has highlighted that differences in water-holding capacity among different starches can be attributed to the influence of the amylose/amylopectin ratio [39]. T_23_ and A_23_ values for NG100, NG82, and NG64 were 0% (Table 3), indicating that water was tightly connected to the starch, leading to a small proportion of free water [40]. As amylose content increased in NG46, NG28, and N010, A_23_ gradually increased. Studies suggest that higher A_23_ values correlate with poorer water-holding capacity, consistent with the WHC results in Table 3. As the proportion of LACCS increased, free water gradually decreased, possibly due to differences in the hydrogen bond structures of amylopectin and amylose. The hydrogen bonding of inter-amylopectin helices could form junction zones, causing free water to enter the spiral cavity, where it becomes fixed and transforms into immobile water and bound water [41].

### 3.6. Starch Distribution by Light Microscopy

**Figure 5:**
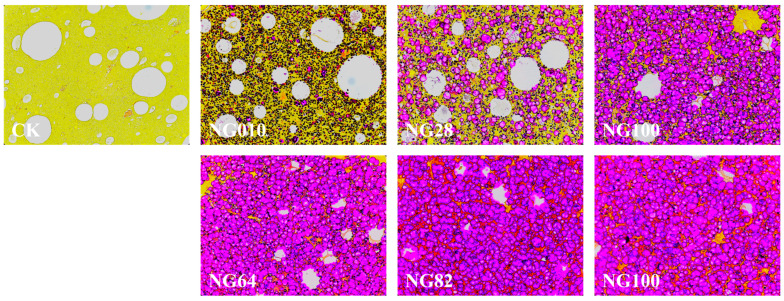
Light microscopy images (10×) with periodic acid-Schiff/naphthol yellow S double-staining of surimi/starch composite gels. NG010, NG28, NG46, NG64, NG82, and NG100 represent surimi/starch composite gels with added mixed starch at mass ratios of LACCS and HACCS at 0:10, 2:8, 4:6, 6:4, 8:2, and 10:0, respectively.

Comprehensive insight into starch behavior within the surimi gel matrix is essential, particularly concerning their dynamics of swelling and diffusion. The distribution and morphology of starch granules in the surimi gel were meticulously examined using light microscopy with periodic acid-Schiff/naphthol yellow S double staining, as depicted in Figure 5. Staining results illuminated the irregular distribution structure of CK, characterized by numerous large and small holes across all surimi gel samples. Upon the addition of starches, a distinct two-phase system emerged. The purple color represents the separate phase formed by starch granules, while the yellow color represents the continuous phase constituted by surimi protein. The uniform dispersion of starch granules without forming large aggregates underscored the limited compatibility between protein and starch [42]. Notably, a high proportion of HACCS in NG010 and NG28 demonstrated minimal expansion, with a notable presence of yellow-colored protein. This may be attributed to the hindered gelatinization of HACCS during heating, resulting in a weaker “packing effect” on the surimi gel [43]. The smaller size of HACCS granules had a lesser impact on the gel structure, promoting a more intact protein network formation and consequently enhancing the strength of the surimi gel [35,44]. With increasing the proportion of LACCS in the surimi/starch composite gels, the number and size of holes in the surimi gel gradually diminished, eventually yielding a smooth, homogeneous, and compact structure (NG64, NG82, and NG100). This finding indicated that corn amylose, with limited water-absorbing capacity, remained relatively inert within the gel network. In contrast, corn amylopectin granules exhibited significant swelling, reinforcing the gel structure and actively filling the gel network [45]. Our study corroborates the concept that the role of starches in the surimi gel matrix aligns with the “packing effect” theory. The overall gel network structure is supported by the expansion of corn amylopectin granules during heating [46].

### 3.7. Microstructure by Cryo-SEM

**Figure 6:**
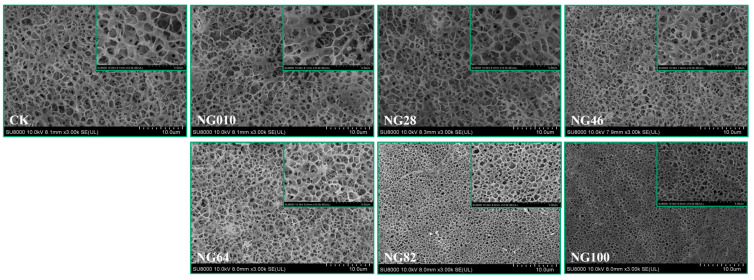
Cryo-SEM (3× and 10×) images of different surimi/starch composite gels. NG010, NG28, NG46, NG64, NG82, and NG100 denote surimi/starch composite gels with added mixed starch at mass ratios of LACCS and HACCS at 0:10, 2:8, 4:6, 6:4, 8:2, and 10:0, respectively.

Figure 6 vividly portrays the microstructure of the surimi gel, a pivotal factor influencing its overall quality and water-holding capacity [47], through cryo-scanning electron microscopy (Cryo-SEM) micrographs. CK exhibits a coarse network structure characterized by sizable cavities and loose organization, consistent with the observations from stained sections in Figure 5. With the incorporation of starches into the surimi matrix, a more compact and porous gel network emerges. Starches function as fillers, creating pressure, imparting rigidity, and establishing water retention spaces within the protein network. This architectural enhancement led to improved texture properties and increased water-holding capacity in surimi/starch composite gels [44,48]. Notably, the NG010 surimi gel sample showed more delicate network structures and larger pore diameters compared to other composite gels. As the proportion of LACCS to starches increased, LACCS expanded and absorbed water, filling the surimi gel and producing a finer gel network characterized by the highest pore density and smallest pore diameter, as corroborated by double staining observations in Figure 5. The reduction in pore size not only bolstered bonding between protein and additive compositions but also fostered cross-linking between proteins, promoting gel rigidity and yielding a more robust network structure [35,49]. Consequently, starches with a higher corn amylopectin content, especially NG82 and NG100, played a pivotal role in shaping a more homogeneous and compact surimi gel network by minimizing pore diameters and maximizing pore density.

### 3.8. Protein Secondary Structure

**Figure 7:**
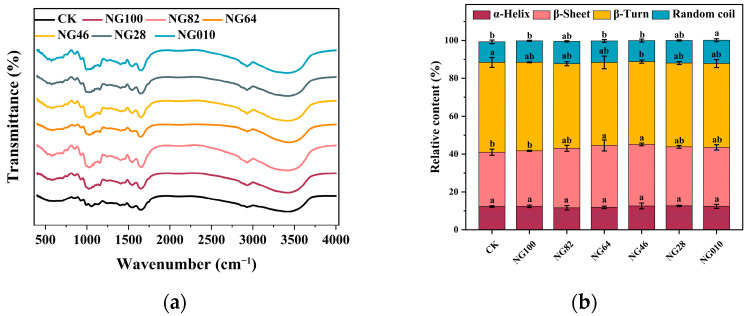
FT-IR (**a**) and relative content (%) of protein secondary structures (**b**) of different surimi/starch composite gels. NG010, NG28, NG46, NG64, NG82, and NG100 represent surimi/starch composite gels with added mixed starch at mass ratios of LACCS and HACCS at 0:10, 2:8, 4:6, 6:4, 8:2, and 10:0, respectively. Different lowercase letters represent the significant difference of surimi/starch composite gels with protein secondary structure content (*p* < 0.05).

In examining the complex gel matrix, FT-IR spectroscopy was employed to discern the functional groups associated with intramolecular and intermolecular structures [50]. The amide I band, encompassing α-helix, random coil, β-sheet, and β-turn structures within the 1650–1660 cm^−1^, 1660–1665 cm^−1^, 1665–1680 cm^−1^, and >1680 cm^−1^ ranges, was a focal point of our analysis [51]. The FT-IR spectroscopy of the surimi gels within the 400–4000 cm^−1^ range is illustrated in Figure 7a, while Figure 7b quantitatively analyzes the protein secondary structure derived from the Amide I spectra. Noteworthy is the elevated relative content of β-turn structures, underscoring their predominant role in the secondary structure of the surimi gel, irrespective of the addition of corn starch or variations in the HACCS to LACCS ratio. Previous studies have emphasized that starch induces minimal shifts in amide bands [20,52]. While starch enhances the gel matrix and influences chemical interactions, its impact on the three-dimensional structure of proteins remains marginal. Subtle modifications in α-helix and random coil structures were observed in surimi gels, fostering an increase in hydrogen bonds. The stability of α-helix in native and partially denatured proteins, coupled with the formation of β structures during heating and cooling, relies on hydrogen bonds [53]. Consequently, the secondary structure of surimi protein remained largely unaffected by external physical forces exerted by starch.

### 3.9. Relationships between Structure and Properties

**Figure 8:**
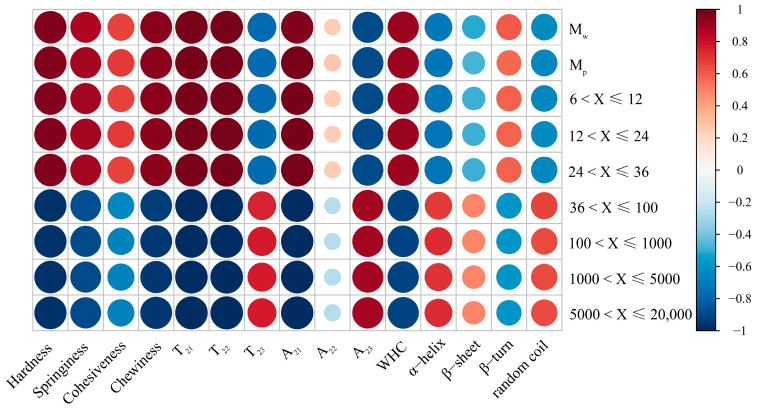
Heat map of the distribution of correlation coefficients between starch molecular structure and properties of different surimi/starch composite gels.

The outcomes of the correlation analyses between starch molecular structure and surimi gel properties are depicted in Figure 8 and Table S1. M_w_ and M_p_ of starch, along with the distribution of chains in different regions (A, B_1_, and B_2_), exhibited robust positive correlations with the hardness and chewiness correlated with the surimi gel. These findings parallel those reported by Yang et al. [54], who explored the interplay between starch molecular structure and cake batter texture properties. One plausible explanation is that the substantial size of starch molecules results in a limited specific surface area, diminishing their capacity to bind with other components or ingredients and consequently augmenting the system’s hardness. Contrary to earlier theories regarding the connection between the texture of cooked rice and starch molecular structure, their findings indicate a strong positive correlation between the distribution of chains in different regions (A, B_1_, and B_2_ of amylopectin) and the hardness and chewiness of the surimi gel. This deviation from past theories may stem from the unique properties of corn starch used in this experiment, which differs significantly from rice starch. During the surimi gelation process, the maximum heating temperature (90 °C) did not reach the gelation temperature of HACCS, and HACCS did not undergo gelatinization, as evidenced in Figure 5. Consequently, the observed change in surimi gel hardness was primarily attributed to the volume expansion of corn amylopectin (A, B_1_, and B_2_) after water absorption. The expanded corn amylopectin then filled the surimi gel, fostering the creation of a denser protein network, as illustrated in Figure 5 and Figure 6. Additionally, chewiness in the surimi gel demonstrated a positive correlation with hardness, aligning with the findings of previous studies [20,55]. The T_21_, T_22_, and A_21_ of the surimi gel showed strong positive correlations with the number of chains in different regions (A, B_1_, and B_2_). Vamadevan et al. [34] postulated that helical hydrogen bonds in amylopectin (A, B_1_, and B_2_) might create a connecting region, allowing free water to penetrate the helical cavity, transforming into bound and immobile water. Interestingly, no discernible relationship was found between the protein secondary structure and the molecular structure of corn starch. This suggests that the amide band remains largely unaffected by the molecular structure of corn starch, consistent with previous research [20,51].

Employing a cluster analysis on diverse surimi/starch composite gels, the cluster trend diagram, as depicted in Figure S1, identifies NG100 and NG28 in a distinct group characterized by augmented bound water, immobile water content, and denser gel networks. This grouping correlates with higher texture attributes such as hardness, springiness, and chewiness. Notably, G100 and NG28, with a higher concentration of A, B_1_, and B_2_, emerge as optimal choices for the food industry’s pursuit of developing surimi gels that are dense, chewy, and possess superior water-retaining properties.

## 4. Conclusions

This study employed size-exclusion and gel-permeation chromatography techniques to scrutinize the molecular structure of corn starch. Complementary methodologies, including texture profile analysis, nuclear magnetic resonance analysis, scanning electron microscopy, stained section examination, and Fourier transform infrared spectroscopy, were leveraged to assess the quality of surimi gel products. Furthermore, correlations were established between corn starch molecular structures and surimi gel properties, encompassing texture profiles, moisture distribution, and protein secondary structure. The findings unveiled an initial surge followed by a subsequent decline in the hardness and chewability of surimi gel as the ratio of LACCS to HACCS increased. Moreover, an enhancement in WHC and bound water content of surimi gel was observed. Despite no significant change in the volume of corn amylose upon water absorption, corn amylopectin (A, B_1_, and B_2_) underwent substantial volume expansion, effectively filling the surimi gel matrix and facilitating the formation of a denser protein network. Furthermore, the M_w_ and M_p_ of starch, along with the chain distribution in different regions (A, B_1_, and B_2_), exhibited robust positive correlations with the hardness and chewiness of the surimi gel. The number of chains in different regions (A, B_1_, and B_2_) demonstrated strong positive associations with T_21_, T_22_, and A_21_ values observed for the surimi gel, favoring the formation of more bound water in the gel. Notably, the protein secondary structure within the surimi gel was found to be unrelated to the molecular structure of corn starch. NG100 and NG28 stand out as prime candidates for crafting surimi gels with desirable characteristics, namely, density, chewiness, and effective water retention. In conclusion, this study furnishes valuable insights into the selection of appropriate corn starch materials for surimi gels, thus advancing the utilization of corn starch materials in the food industry.

## Figures and Tables

**Table 1:** Molecular structure parameters of whole starch samples. Mw, weight-average molecular weight: Mp, peak molecular weight; AM, amylose; AP, amylopectin; DP, degree of polymerization. NG010, NG28, NG46, NG64, NG82, and NG100 denote the mixed starch samples with mass ratio of LACCS and HACCS at 0:10, 2:8, 4:6, 6:4, 8:2, and 10:0, respectively.

**Samples**	**Structure Parameters**										
	**Mw**		**Mp**			**Amylose**		**Amylopectin**		**AM/AP**	
NG100	65,304.72 ± 1704.51 ^a^		113,771.44 ± 9508.12 ^a^			6.44 ± 0.37 ^f^		93.56 ± 0.37 ^a^		0.07 ± 0.00 ^f^	
NG82	52,146.74 ± 1642.80 ^b^		93,568.84 ± 2904.67 ^b^			19.55 ± 0.76 ^e^		80.45 ± 0.76 ^b^		0.24 ± 0.01 ^e^	
NG64	39,400.75 ± 1452.86 ^c^		71,146.25 ± 3697.08 ^c^			32.34 ± 1.72 ^d^		67.66 ± 1.72 ^c^		0.48 ± 0.04 ^d^	
NG46	26,794.76 ± 686.66 ^d^		47,966.99 ± 1300.05 ^d^			45.25 ± 2.60 ^c^		54.75 ± 2.60 ^d^		0.83 ± 0.09 ^c^	
NG28	14,578.11 ± 467.40 ^e^		24,154.40 ± 1127.36 ^e^			58.73 ± 1.16 ^b^		41.27 ± 1.16 ^e^		1.42 ± 0.07 ^b^	
NG010	1893.79 ± 73.25 ^f^		1331.81 ± 102.23 ^f^			69.89 ± 1.31 ^a^		30.11 ± 1.31 ^f^		2.33 ± 0.14 ^a^	
**Samples**	**DP**										
	**6 < X ≤ 12** **(A)**	**12 < X ≤ 24** **(B_1_** **)**		**24 < X ≤ 36** **(B_2_** **)**	**36 < X ≤ 100** **(B_3_** **)**		**100 < X ≤ 1000**		**1000 < X ≤ 5000**		**5000 < X ≤ 20,000**
NG100	28.42 ± 2.16 ^a^	33.94 ± 2.58 ^a^		14.53 ± 0.70 ^a^	16.29 ± 0.44 ^d^		1.26 ± 0.20 ^f^		0.17 ± 0.02 ^f^		0.25 ± 0.01 ^f^
NG82	23.49 ± 1.51 ^b^	29.04 ± 1.55 ^b^		12.94 ± 0.25 ^b^	16.96 ± 0.09 ^cd^		8.15 ± 0.25 ^e^		4.45 ± 0.24 ^e^		1.07 ± 0.07 ^e^
NG64	18.61 ± 1.81 ^c^	23.79 ± 0.91 ^c^		11.34 ± 0.23 ^c^	17.55 ± 1.18 ^bcd^		14.56 ± 1.72 ^d^		8.72 ± 0.33 ^d^		1.85 ± 0.22 ^d^
NG46	13.91 ± 0.96 ^d^	18.56 ± 1.06 ^d^		9.71 ± 0.11 ^d^	18.02 ± 0.74 ^abc^		21.84 ± 0.42 ^c^		12.95 ± 0.26 ^c^		2.72 ± 0.04 ^c^
NG28	9.01 ± 0.13 ^e^	13.40 ± 0.68 ^e^		8.10 ± 0.10 ^e^	18.77 ± 0.59 ^ab^		28.42 ± 1.76 ^b^		17.25 ± 0.44 ^b^		3.52 ± 0.03 ^b^
NG010	4.19 ± 0.36 ^f^	8.32 ± 0.42 ^f^		6.48 ± 0.02 ^f^	19.30 ± 1.03 ^a^		35.25 ± 2.15 ^a^		21.54 ± 0.84 ^a^		4.36 ± 0.11 ^a^

Data are expressed as means ± SD from triplicate determinations. Values followed by different superscript letters in the same column are considered significantly different (*p* < 0.05).

**Table 2:** Texture analysis of different surimi/starch composite gels. NG010, NG28, NG46, NG64, NG82, and NG100 represent the surimi/starch composite gels with added mixed starch, where the mass ratio of LACCS and HACCS is 0:10, 2:8, 4:6, 6:4, 8:2, and 10:0, respectively.

Sample	Texture Analysis			
	Hardness (g)	Springiness	Cohesiveness	Chewiness
CK	1028.54 ± 11.75 ^g^	0.85 ± 0.00 ^e^	0.70 ± 0.00 ^a^	615.33 ± 8.38 ^g^
NG100	2357.44 ± 16.62 ^b^	0.90 ± 0.01 ^b^	0.68 ± 0.01 ^c^	1439.05 ± 15.62 ^b^
NG82	2438.10 ± 35.28 ^a^	0.92 ± 0.00 ^a^	0.69 ± 0.00 ^b^	1559.55 ± 23.15 ^a^
NG64	2105.61 ± 21.20 ^c^	0.90 ± 0.00 ^b^	0.68 ± 0.00 ^c^	1267.58 ± 28.23 ^c^
NG46	1933.97 ± 34.39 ^d^	0.89 ± 0.01 ^c^	0.68 ± 0.00 ^c^	1145.99 ± 5.31 ^d^
NG28	1759.47 ± 9.52 ^e^	0.87 ± 0.00 ^d^	0.68 ± 0.00 ^cd^	1051.32 ± 3.77 ^e^
NG010	1534.18 ± 18.65 ^f^	0.85 ± 0.00 ^e^	0.67 ± 0.00 ^d^	862.51 ± 4.76 ^f^

Data are expressed as means ± SD from triplicate determinations. Values followed by different superscript letters in the same column are considered significantly different (*p* < 0.05).

**Table 3:** LF-NMR spin-spin relaxation time (T_2_), peak area percentage of the three populations, and WHC of surimi/starch composite gels. NG010, NG28, NG46, NG64, NG82, and NG100 represent the surimi/starch composite gels with added mixed starch, where the mass ratio of LACCS and HACCS is 0:10, 2:8, 4:6, 6:4, 8:2, and 10:0, respectively.

Sample	WHC (%)	T_2_ (ms)			A_2_ (%)		
		T_21_	T_22_	T_23_	A_21_	A_22_	A_23_
CK	69.50 ± 0.44 ^e^	1.79 ± 0.15 ^f^	123.22 ± 4.88 ^a^	822.75 ± 57.09 ^a^	0.60 ± 0.03 ^a^	97.22 ± 0.28 ^a^	2.66 ± 0.11 ^b^
NG100	93.16 ± 0.06 ^a^	10.37 ± 0.72 ^a^	120.41 ± 4.88 ^a^	-	5.75 ± 0.06 ^b^	94.15 ± 0.13 ^c^	-
NG82	92.89 ± 0.18 ^a^	9.46 ± 0.77 ^b^	109.70 ± 0.00 ^b^	-	5.42 ± 0.09 ^c^	94.48 ± 0.10 ^c^	-
NG64	90.12 ± 0.21 ^b^	8.42 ± 0.59 ^c^	100.05 ± 3.96 ^c^	-	4.77 ± 0.12 ^d^	95.08 ± 0.08 ^b^	-
NG46	90.07 ± 0.26 ^b^	5.55 ± 0.39 ^d^	83.23 ± 5.77 ^d^	682.97 ± 27.68 ^b^	3.94 ± 0.08 ^e^	94.97 ± 0.21 ^b^	0.80 ± 0.04 ^d^
NG28	86.14 ± 0.33 ^c^	4.21 ± 0.29 ^e^	67.59 ± 4.69 ^e^	517.37 ± 20.98 ^c^	2.54 ± 0.11 ^f^	94.94 ± 0.08 ^b^	2.38 ± 0.11 ^c^
NG010	78.86 ± 1.16 ^d^	2.59 ± 0.18 ^f^	54.88 ± 3.81 ^f^	450.30 ± 18.26 ^d^	1.62 ± 0.07 ^g^	93.23 ± 0.33 ^d^	4.64 ± 0.11 ^a^

Data are expressed as means ± SD from triplicate determinations. Values followed by different superscript letters in the same column are considered significantly different (*p* < 0.05).

## Data Availability

The original contributions presented in the study are included in the article/Supplementary Materials, further inquiries can be directed to the corresponding author.

## Supplementary Materials

The following supporting information can be downloaded at https://www.mdpi.com/article/10.3390/foods13050675/s1. Figure S1: Cluster trend diagram of different surimi/starch composite gels; Table S1: Correlation coefficients between starch structure and surimi/starch composite gels; Table S2: Protein secondary structures percentage of different surimi/starch composite gels.

## References

[1] Alipour H.J., Rezaei M., Shabanpour B., Tabarsa M. (2018). Effects of sulfated polysaccharides from green alga Ulva intestinalis on physicochemical properties and microstructure of silver carp surimi. Food Hydrocoll..

[2] Sun F., Huang Q., Hu T., Xiong S., Zhao S. (2014). Effects and mechanism of modified starches on the gel properties of myofibrillar protein from grass carp. Int. J. Biol. Macromol..

[3] Kok N., Thawornchinsombut S., Park J.W. (2014). Manufacture of surimi. Surimi and Surimi Seafood.

[4] Kong W., Zhang T., Feng D., Xue Y., Wang Y., Li Z., Yang W., Xue C. (2016). Effects of modified starches on the gel properties of Alaska Pollock surimi subjected to different temperature treatments. Food Hydrocoll..

[5] Liu H., Nie Y., Chen H. (2014). Effect of different starches on colors and textural properties of surimi-starch gels. Int. J. Food Prop..

[6] Jia R., Katano T., Yoshimoto Y., Gao Y., Nakazawa N., Osako K., Okazaki E. (2020). Effect of small granules in potato starch and wheat starch on quality changes of direct heated surimi gels after freezing. Food Hydrocoll..

[7] Kim J.M., Lee C.M. (1987). Effect of starch on textural properties of surimi gel. J. Food Sci..

[8] Tappiban P., Sraphet S., Srisawad N., Wu P., Han H., Smith D.R., Bao J., Triwitayakorn K. (2020). Effects of cassava variety and growth location on starch fine structure and physicochemical properties. Food Hydrocoll..

[9] Kong X., Bertoft E., Bao J., Corke H. (2008). Molecular structure of amylopectin from amaranth starch and its effect on physico-chemical properties. Int. J. Biol. Macromol..

[10] Li G., Zhu F. (2018). Rheological properties in relation to molecular structure of quinoa starch. Int. J. Biol. Macromol..

[11] Du J., Pan R., Obadi M., Li H., Shao F., Sun J., Wang Y., Qi Y., Xu B. (2022). In vitro starch digestibility of buckwheat cultivars in comparison to wheat: The key role of starch molecular structure. Food Chem..

[12] Tao K., Yu W., Prakash S., Gilbert R.G. (2019). High-amylose rice: Starch molecular structural features controlling cooked rice texture and preference. Carbohyd. Polym..

[13] Ekonomou S.Ι., Hadnađev M., Gioxari A., Abosede O.R., Soe S., Stratakos A.C. (2024). Advancing dysphagia-oriented multi-ingredient meal development: Optimising hydrocolloid incorporation in 3D printed nutritious meals. Food Hydrocoll..

[14] Cortez-Trejo M.C., Gaytán-Martínez M., Reyes-Vega M.L., Mendoza S. (2021). Protein-gum-based gels: Effect of gum addition on microstructure, rheological properties, and water retention capacity. Trends Food Sci. Technol..

[15] Kang X., Jia S., Gao W., Wang B., Zhang X., Tian Y., Sun Q., Atef M., Cui B., Abd El-Aty A.M. (2022). The formation of starch-lipid complexes by microwave heating. Food Chem..

[16] Pan L., Chen F., Yang Y., Li Q., Fan X., Zhao D., Liu Q., Zhang C. (2022). The underlying starch structures of rice grains with different digestibilities but similarly high amylose contents. Food Chem..

[17] Hanashiro I., Abe J., Hizukuri S. (1996). A periodic distribution of the chain length of amylopectin as revealed by high-performance anion-exchange chromatography. Carbohyd. Res..

[18] Rice-Determination of Amylose Content.

[19] Luo H., Guo C., Lin L., Si Y., Gao X., Xu D., Jia R., Yang W. (2020). Combined use of rheology, LF-NMR, and MRI for characterizing the gel properties of hairtail surimi with potato starch. Food Bioprocess Technol..

[20] Zhao Y., Wei G., Li J., Tian F., Zheng B., Gao P., Zhou R. (2023). Comparative study on the effect of different salts on surimi gelation and gel properties. Food Hydrocoll..

[21] Piao X., Huang J., Sun Y., Zhao Y., Zheng B., Zhou Y., Yu H., Zhou R., Cullen P.J. (2023). Inulin for surimi gel fortification: Performance and molecular weight-dependent effects. Carbohyd. Polym..

[22] Sun X., Lv Y., Jia H., Mráz J., Gu Y., Xu X., Li S., Dong X., Pan J. (2024). Improvement of flavor and gel properties of silver carp surimi product by Litsea cubeba oil high internal phase emulsions. LWT-Food Sci..

[23] Zhao X., Wang X., Zeng L., Huang Q., Zhang J., Wen X., Xiong S., Yin T., Zhang B. (2022). Effects of oil-modified crosslinked/acetylated starches on silver carp surimi gel: Texture properties, water mobility, microstructure, and related mechanisms. Food Res. Int..

[24] Zhou X., Jiang S., Zhao D., Zhang J., Gu S., Pan Z., Ding Y. (2017). Changes in physicochemical properties and protein structure of surimi enhanced with camellia tea oil. LWT-Food Sci. Technol..

[25] Jiang X., Chen Q., Xiao N., Du Y., Feng Q., Shi W. (2021). Changes in Gel Structure and Chemical Interactions of Hypophthalmichthys molitrix Surimi Gels: Effect of Setting Process and Different Starch Addition. Foods.

[26] Li C., Gong B. (2021). Relations between rice starch fine molecular and lamellar/crystalline structures. Food Chem..

[27] Shi Y.-C., Capitani T., Trzasko P., Jeffcoat R. (1998). Molecular structure of a low-amylopectin starch and other high-amylose maize starches. J. Cereal Sci..

[28] Yang F., Zhang M., Bhandari B., Liu Y. (2018). Investigation on lemon juice gel as food material for 3D printing and optimization of printing parameters. LWT-Food Sci. Technol..

[29] Alakhrash F., Anyanwu U., Tahergorabi R. (2016). Physicochemical properties of Alaska pollock (*Theragra chalcograma*) surimi gels with oat bran. LWT-Food Sci. Technol..

[30] Mi H., Wang C., Su Q., Li X., Yi S., Li J. (2019). The effect of modified starches on the gel properties and protein conformation of Nemipterus virgatus surimi. J. Texture Stud..

[31] Friedman H.H., Whitney J.E., Szczesniak A.S. (1963). The texturometer-A new instrument for objective texture measurement. J. Food Sci..

[32] Wu W., Que F., Li X., Shi L., Deng W., Fu X., Xiong G., Sun J., Wang L., Xiong S. (2022). Effects of enzymatic konjac glucomannan hydrolysates on textural properties, microstructure, and water distribution of grass carp surimi gels. Foods.

[33] Mi H., Su Q., Chen J., Yi S., Li X., Li J. (2021). Starch-fatty acid complexes improve the gel properties and enhance the fatty acid content of Nemipterus virgatus surimi under high-temperature treatment. Food Chem..

[34] Vamadevan V., Bertoft E. (2020). Observations on the impact of amylopectin and amylose structure on the swelling of starch granules. Food Hydrocoll..

[35] Pereira J., Malairaj S., Brohi S.A., Boateng E.F., Zhang W. (2020). Impact of unripe banana flour on water states, rheological behaviour and structural properties of myofibrillar protein composite gel. LWT-Food Sci. Technol..

[36] Dong M., Tian H., Xu Y., Han M. (2020). Effects of pulsed electric fields on the conformation and gelation properties of myofibrillar proteins isolated from pale, soft, exudative (PSE)-like chicken breast meat: A molecular dynamics study. Food Chem..

[37] Zhao X., Wang X., Li X., Zeng L., Huang J., Huang Q., Zhang B. (2022). Effect of oil modification on the multiscale structure and gelatinization properties of crosslinked starch and their relationship with the texture and microstructure of surimi/starch composite gels. Food Chem..

[38] Zhang L., Xue Y., Xu J., Li Z. (2013). Effects of high-temperature treatment (≥100 °C) on Alaska Pollock (*Theragrachalcogramma*) surimi gels. J. Food Eng..

[39] Wootton M., Bamunuarachchi A. (1978). Water binding capacity of commercial produced native and modified starches. Starch-Stärke.

[40] Ghazal A.F., Zhang M., Bhandari B., Chen H. (2021). Investigation on spontaneous 4D changes in color and flavor of healthy 3D printed food materials over time in response to external or internal pH stimulus. Food Res. Int..

[41] Zhou X., Wang R., Yoo S.H., Lim S.T. (2011). Water effect on the interaction between amylose and amylopectin during retrogradation. Carbohyd. Polym..

[42] Chen D., Fang F., Federici E., Campanell O. (2020). Rheology, microstructure and phase behavior of potato starch-protein fibril mixed gel. Carbohyd. Polym..

[43] Wang X., Liu S., Ai Y. (2022). Gelation mechanisms of granular and non-granular starches with variations in molecular structures. Food Hydrocoll..

[44] Mi H., Li Y., Wang C., Yi S. (2021). The interaction of starch-gums and their effect on gel properties and protein conformation of silver carp surimi. Food Hydrocoll..

[45] Yang H., Park J.W. (1998). Effects of starch properties and thermal-processing conditions on surimi–starch gels. LWT-Food Sci. Technol..

[46] Wu M., Wang G., Ge Q., Yu H. (2018). Rheology and microstructure of myofibrillar protein–starch composite gels: Comparison of native and modified starches. Int. J. Biol. Macromol..

[47] Fan M., Huang Q., Zhong S., Li X., Xiong S., Xie J., Yin T., Zhang B., Zhao S. (2019). Gel properties of myofibrillar protein as affected by gelatinization and retrogradation behaviors of modified starches with different crosslinking and acetylation degrees. Food Hydrocoll..

[48] Wang X., Li Y., Zhou Y., Ma F. (2019). Effect of resistant corn starch on the thermal gelling properties of chicken breast myosin. Food Hydrocoll..

[49] Yang Z., Wang W., Wang H., Ye Q. (2014). Effects of a highly resistant rice starch and pre-incubation temperatures on the physicochemical properties of surimi gel from grass carp (*Ctenopharyn Odon Idellus*). Food Chem..

[50] Yu W., Wang Z., Pan Y., Jiang P., Pan J., Yu C., Dong X. (2022). Effect of κ-carrageenan on quality improvement of 3D printed Hypophthalmichthys molitrix-sea cucumber compound surimi product. LWT-Food Sci. Technol..

[51] Zhuang X., Jiang X., Zhou H., Chen Y., Zhao Y., Yang H., Zhou G. (2020). Insight into the mechanism of physicochemical influence by three polysaccharides on myofibrillar protein gelation. Carbohyd. Polym..

[52] Zhang L., Li Q., Shi J., Zhu B., Luo Y. (2018). Changes in chemical interactions and gel properties of heat-induced surimi gels from silver carp (*Hypophthalmichthys molitrix*) fillets during setting and heating: Effects of different washing solutions. Food Hydrocoll..

[53] Park J.W., Lin T.-M., Yongsawatdigul J. (1997). New developments in manufacturing of surimi and surimi seafood. Food Rev. Int..

[54] Yang X., Pan Y., Li S., Li C., Li E. (2022). Effects of amylose and amylopectin molecular structures on rheological, thermal and textural properties of soft cake batters. Food Hydrocoll..

[55] Petcharat T., Benjakul S. (2018). Effect of gellan incorporation on gel properties of bigeye snapper surimi. Food Hydrocoll..

