# Supplementary material for: Impact of Corn Starch Molecular Structures on Texture, Water Dynamics, Microstructure, and Protein Structure in Silver Carp (Hypophthalmichthys molitrix) Surimi Gel
Source: Foods. 2024 Feb 23;13(5):675. doi: 10.3390/foods13050675 (PMC10931399; doi:10.3390/foods13050675)
Supplement: Supplementary file 1 [file foods-13-00675-s001.zip › foods-2853229-supplementary.pdf]

Table S1. Correlation coefficients between starch structure and surimi/starch composite gels.

	M_w	M_p	6 < X ≤ 12 (A)	12 < X ≤ 24 (B_1)	24 < X ≤ 36 (B_2)	36 < X ≤ 100 (B_3)	100 < X ≤ 1000	1000 < X ≤ 5000	5000 < X ≤ 20000
Hardness	0.968**	0.974**	0.969**	0.972**	0.970**	-0.964**	-0.969**	-0.970**	-0.970**
Springiness	0.878*	0.893*	0.881*	0.886*	0.884*	-0.877*	-0.882*	-0.883*	-0.884*
Cohesivene	0.672	0.687	0.674	0.680	0.678	-0.660	-0.673	-0.676	-0.676
Chewiness	0.944**	0.951**	0.945**	0.949**	0.947**	-0.937**	-0.945**	-0.946**	-0.946**
T_{21}	0.987**	0.990**	0.987**	0.989**	0.988**	-0.982**	-0.989**	-0.988**	-0.989**
T_{22}	0.995**	0.998**	0.996**	0.996**	0.996**	-0.994**	-0.996**	-0.996**	-0.997**
T_{23}	-0.772	-0.773	-0.769	-0.775	-0.773	0.748	0.778	0.770	0.776
A_{21}	0.979**	0.985**	0.980**	0.982**	0.981**	-0.981**	-0.981**	-0.981**	-0.982**
A_{22}	0.247	0.267	0.251	0.252	0.255	-0.246	-0.255	-0.254	-0.259
A_{23}	-0.882*	-0.895*	-0.885*	-0.887*	-0.887*	0.886*	0.887*	0.887*	0.889*
WHC	0.910*	0.918**	0.912*	0.913*	0.913*	-0.913*	-0.911*	-0.913*	-0.913*
α-helix	-0.713	-0.734	-0.715	-0.724	-0.721	0.697	0.722	0.718	0.723
β-sheet	-0.505	-0.474	-0.498	-0.491	-0.493	0.493	0.493	0.495	0.491
β-turn	0.602	0.574	0.597	0.590	0.591	-0.597	-0.593	-0.593	-0.589
Random	-0.637	-0.648	-0.642	-0.639	-0.642	0.660	0.641	0.643	0.643

* and ** indicate significance at $p < 0.05$ and 0.01, respectively.

Table S2. Protein secondary structures percentage of different surimi/starch composite gels. NG010, NG28, NG46, NG64, NG82, NG100 represent the surimi/starch composite gels with added mixed starch, where the mass ratio of LACCS and HACCS is 0:10, 2:8, 4:6, 6:4, 8:2, and 10:0, respectively.

Sample	Protein secondary structures content (%)			
	α-helix	β-sheet	β-turn	random coil
CK	12.28 ± 0.41^{a}	29.49 ± 1.62^{b}	47.36 ± 2.60^{a}	10.87 ± 0.96^{b}
NG100	12.39 ± 0.64^{a}	29.37 ± 0.34^{b}	46.67 ± 0.21^{ab}	11.36 ± 0.29^{b}
NG82	11.61 ± 1.17^{a}	31.52 ± 1.58^{ab}	44.57 ± 1.06^{ab}	11.83 ± 0.33^{ab}
NG64	11.86 ± 0.67^{a}	32.76 ± 2.91^{a}	43.75 ± 3.32^{ab}	11.39 ± 0.73^{b}
NG46	12.58 ± 1.55^{a}	32.58 ± 0.71^{a}	43.64 ± 0.82^{b}	11.12 ± 0.79^{b}
NG28	12.64 ± 0.36^{a}	31.25 ± 0.75^{ab}	44.14 ± 0.78^{ab}	11.96 ± 0.32^{ab}
NG010	13.08 ± 1.06^{a}	31.83 ± 1.40^{ab}	44.49 ± 2.10^{ab}	12.72 ± 0.77^{a}

Data are expressed as means ± SD from triplicate determinations. Values followed by different superscript letters in the same column are considered significantly different ($p < 0.05$).

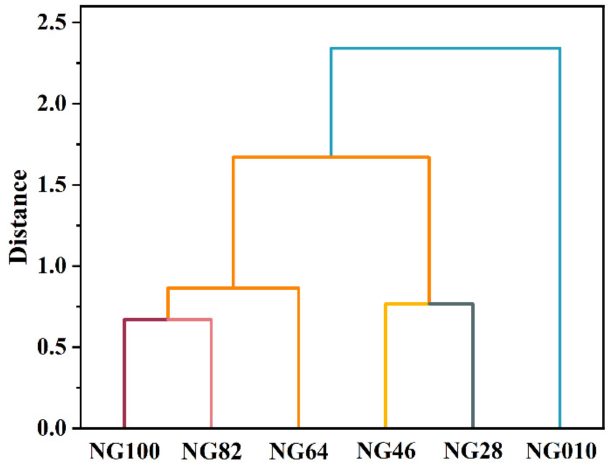

Figure S1. Cluster trend diagram of different surimi/starch composite gels. NG010, NG28, NG46, NG64, NG82, NG100 represent surimi/starch composite gels with added mixed starch at mass ratios of LACCS and HACCS at 0:10, 2:8, 4:6, 6:4, 8:2, and 10:0, respectively
